# Complete Genome Sequence of Staphylococcus aureus Phage SA75, Isolated from Goat Feces

**DOI:** 10.1128/MRA.00114-20

**Published:** 2020-04-16

**Authors:** Roshan D’Souza, Richard C. White, Rachel Buzzeo, Karrie Goglin, Sanjay Vashee, Yoona Lee, Bokyung Son, Sangryeol Ryu, Derrick E. Fouts

**Affiliations:** a J. Craig Venter Institute, Rockville, Maryland, USA; b J. Craig Venter Institute, La Jolla, California, USA; c Department of Food and Animal Biotechnology, Department of Agricultural Biotechnology, Seoul National University, Seoul, South Korea; DOE Joint Genome Institute

## Abstract

Antibiotic-resistant Staphylococcus aureus is an opportunistic pathogen causing serious human infections worldwide. Here, we report the complete annotated genome of bacteriophage SA75, a member of the *Siphoviridae* family which could be an alternative to traditional antibiotics for treating *Staphylococcus* infections. We used a hybrid approach combining MinION and Illumina MiSeq sequencing, which yielded a 43,134-bp genome and 65 open reading frames.

## ANNOUNCEMENT

Staphylococcus aureus, a Gram-positive coccal bacterium, is frequently found in the upper respiratory tract, intestine, and skin; however, it also causes a wide range of infections and food poisoning in humans (1, 2). S. aureus infections are difficult to treat due to an increased rate of resistance to several antibiotics and its ability to quickly adapt to different conditions (3, 4). The use of bacteriophages to treat S. aureus infections and as a biocontrol agent has become an attractive alternative/supplementation to traditional antibiotics and control measures (5–7).

SA75, a novel bacteriophage that infects S. aureus, was isolated from *Capra hircus coreanae* (also known as native Korean goat) feces with S. aureus strain RN4220 as the indicator host strain, using methods previously described (8). Briefly, a fecal sample (25 g) was homogenized in 225 ml of sodium chloride-magnesium sulfate (SM) buffer, mixed with tryptic soy broth (TSB) supplemented with 10 mM CaCl_2_, subcultured with S. aureus RN4220, and incubated at 37°C for 12 h with shaking. After incubation, the samples were centrifuged at 8,000 × *g* for 10 min and filtered to remove bacterial cells and obtain the supernatant containing the bacteriophage. These phages were further plaque purified three times by the agar overlay method to ensure the purity.

For large-scale phage production, TSB was inoculated with S. aureus RN4220, incubated at 37°C for 1.5 h prior to the addition of SA75 at a multiplicity of infection (MOI) of 1, and incubated for 3 h at the same temperature with shaking. The phages were precipitated with polyethylene glycol (PEG) 6000 and concentrated using CsCl density gradient ultracentrifugation. Finally, to confirm viability following CsCl purification, the supernatant was overlaid on 0.4% molten tryptic soy agar (TSA) and S. aureus RN4220. Plaques were evident after incubation at 37°C for 12 h. Bacteriophage genomic DNA was purified as previously described with minor modifications (9). In brief, 500 μl of phage lysate was treated at 37°C for 1 h with 125 U of Benzonase (Sigma), 10 U of recombinant DNase I (rDNase I; Invitrogen), and 10 μl of RNA cocktail solution (Invitrogen) to remove residual S. aureus DNA and RNA. Benzonase was deactivated by the addition of 50 μl of 0.5 M EDTA and 50 μl of 0.5 M EGTA at 70°C for 10 min. Then, 1.6 U of Proteinase K (New England BioLabs, Inc., Ipswich, MA) and 0.5% of SDS were added, and the mix was incubated at 56°C for 1 h, followed by phenol-chloroform DNA extraction.

Illumina sequencing libraries were prepared using the Nextera XT library kit. Sequencing was performed on an Illumina MiSeq instrument using the v2 300-cycle reagent kit, producing 2,055,788 reads with 620.8 million bases. The MinION sequencing libraries were prepared using the rapid barcoding kit (SQK-RBK004) and sequenced using a MinION R9.4 flow cell (Nanopore, Oxford, UK). MinION reads were base called with Albacore software (Nanopore). Illumina read quality was assessed using FastQC v0.11.7, and trimming was performed using Trimmomatic v0.32 (9, 10) with the following settings: ILLUMINACLIP, TruSeq3-PE-2.fa:2:30:10; LEADING, 3; TRAILING, 3; SLIDINGWINDOW, 4:24; and MINLEN, 60. The MinION reads were demultiplexed and quality trimmed using Porechop v0.2.3 (11) with default settings. A hybrid Illumina-MinION *de novo* assembly was performed using the Unicycler v0.4.7 (12) pipeline and corrected for errors using Pilon v1.22 (13) with Illumina reads. The average depth of read coverage for the final assembly, calculated using CLC Genomics Workbench v10.1.1, was 35× and 11,988× for the MinION and Illumina reads, respectively. Automated genome annotation was performed with the Rapid Annotations using Subsystems Technology (RAST) server (13, 14). tRNAscan-SE v2.0 (15) was used to search for tRNAs.

The genome of phage SA75 is 43,134 bp long with 65 predicted open reading frames (ORFs), no tRNAs, and 34.4% GC content. Despite forming clear plaques on RN4220, a lysogeny control region was identified consisting of three genes flanked by an *attP* identical to that of ϕ80α (16), along with an integrase at one end and a *cI*-like repressor at the other end. Although SA75 lacks known phage-encoded S. aureus virulence genes (*lukSF*, *eta*, *sasX*, *sea*, *sep*, *sek*, *seg*, *sak*, *chp*, and *scn*; 17–25), it does encode a dUTPase, which has been shown to facilitate mobilization of S. aureus pathogenicity islands (26, 27). A Web-based MegaBLAST search was performed to identify the closest related phages using default settings (https://blast.ncbi.nlm.nih.gov/Blast.cgi). Average nucleotide identity (ANI) was calculated using the ANI calculator (https://www.ezbiocloud.net/tools/ani; 28), which indicated that SA75 was most closely related to S. aureus *Siphoviridae* phages SA12 (98.98%), SP6 (98.68%), ϕMR25 (95.89%), ϕNM2 (95.77%), and ϕ80α (94.59%). PhageTerm v1.0.12 (29) was unable to identify terminal ends of the phage, suggesting that SA75 was terminally redundant and uses a headful packaging mechanism, which was supported by phylogenetic analysis of the predicted large terminase gene (30).

### Data availability.

The genome sequence of phage SA75 was submitted to GenBank under accession number MT013111. The associated BioProject, SRA, and BioSample accession numbers are PRJNA591820, SRP234516, and SAMN13389916, respectively.
